# Genetic pharmacoepidemiology of JAK inhibitors in chronic immune-mediated skin diseases: implications for precision therapy and medication safety

**DOI:** 10.3389/fphar.2026.1738089

**Published:** 2026-03-19

**Authors:** Yuping Zhang, Yuan Qu, Qundi Luo, Bibo Liang, Qiaomu Jiang, Xiaotong Zhu, Jing Wu, Weijia Bao

**Affiliations:** Department of Rheumatology and Clinical Immunology, Zhujiang Hospital, Southern Medical University, Guangzhou, China

**Keywords:** genetic pharmacoepidemiology, immune-mediated inflammatory skin diseases, janus kinase (JAK) inhibitors, pharmacovigilance and drug safety, summary-data-based mendelian randomization (SMR)

## Abstract

**Background:**

This study aims to investigate the associations between genetically proxied JAK inhibition and various disease outcomes and adverse effects, providing insights into therapeutic efficacy and potential side effects for immune-mediated skin diseases (IMIDs).

**Methods:**

Using Mendelian Randomization (MR), we analyzed genetic proxies for JAK1, JAK2, JAK3, and TYK2 inhibition. Data from the UK Biobank (N = 361,194) and FinnGen (N = 453,733) were utilized to explore associations with nine autoimmune skin diseases, and twelve adverse outcomes, including tuberculosis, non-melanoma skin cancer, lung cancer, and pulmonary embolism. A systematic review of randomized controlled trials (RCTs) on JAK inhibitors in IMIDs was performed to complement the genetic evidence, focusing on safety outcomes.

**Results:**

Summary-data-based MR (SMR) analysis revealed that genetically proxied loss of function mutation of TYK2 was linked to psoriasis (OR = 0.673, 95% CI = 0.512–0.884, p = 0.004), aligning with clinical evidence. Safety analyses yielded genetically supported, hypothesis-generating signals with effect sizes close to unity, including associations between JAK2 inhibition and pulmonary embolism (OR = 0.998, p = 0.035) and tuberculosis (OR = 1.004, p = 0.013), as well as TYK2 inhibition and malignant non-melanoma skin cancer (OR = 1.006, p = 0.047), and lung cancer (OR = 1.002, p = 0.029). These findings should be interpreted cautiously and do not constitute definitive evidence of drug-induced adverse effects. The systematic review partially confirmed the safety of JAK inhibitors in IMIDs.

**Conclusion:**

This study supports TYK2 inhibition as a targeted therapy for psoriasis and provides hypothesis-generating genetic evidence for additional target–disease and target–safety associations that warrant further validation.

## 1 Introduction

Janus kinase (JAK) inhibitors represent a promising class of targeted therapies that have gained significant attention for their role in modulating immune responses. JAK inhibitors have broadened their therapeutic applications to include a range of autoimmune and inflammatory disorders, such as rheumatoid arthritis, ulcerative colitis, and, more recently, dermatological conditions like atopic dermatitis and psoriasis (Benucci et al., 2023; Luo et al., 2021).

The JAK family, consisting of JAK1, JAK2, JAK3, and TYK2 (tyrosine kinase 2), serves as a crucial signaling hub for cytokine receptors. Blocking their activity can broadly regulate inflammatory signaling by affecting interleukins, interferons, and other growth-related cytokines. Clinical trials have demonstrated the efficacy of JAK inhibitors in reducing disease severity in immune-mediated skin diseases (IMIDs) like alopecia areata, vitiligo, and atopic dermatitis (Chapman et al., 2022a; Chapman et al., 2022b). However, despite their promising results, several uncertainties remain regarding their long-term safety, specificity, and off-target effects (Wood et al., 2021). For instance, while JAK inhibitors offer a broad spectrum of cytokine inhibition, this may also lead to unintended immunosuppression and increased susceptibility to infections or malignancies (Martinez et al., 2023).

Moreover, the heterogeneous nature of IMIDs, driven by complex genetic and environmental interactions, adds another layer of complexity to optimizing JAK-targeted therapies. Different patients may exhibit distinct immune profiles and genetic susceptibilities that influence their response to JAK inhibition (Chapman et al., 2022a; Chapman et al., 2022b). Therefore, a one-size-fits-all approach may not be suitable for treating these disorders, and a deeper understanding of the genetic and molecular mechanisms driving disease pathogenesis and treatment response is necessary, particularly in the context of IMIDs (Philips et al., 2022).

**FIGURE 1:**
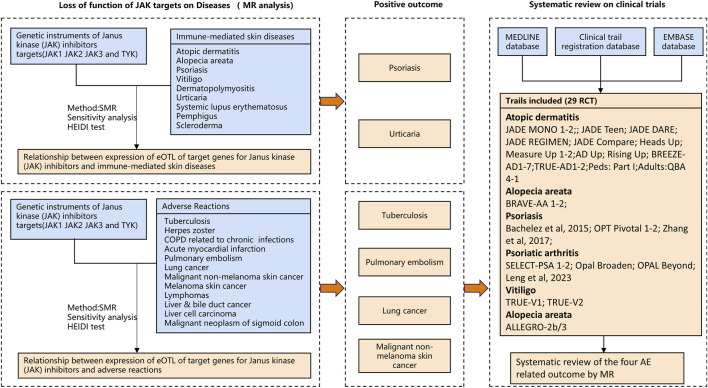
An overview of the study design.

In this context, Mendelian randomization (MR) and summary-data-based MR (SMR) offers a powerful genetic tool to infer causal relationships between genetically proxied perturbation of JAK targets and immune-mediated skin disease outcomes (Davies et al., 2018). Specifically, cis-eQTL-derived instruments can proxy inter-individual variation in the expression of JAK pathway genes, thereby approximating the directional consequences of on-target modulation relevant to pharmacologic inhibition. By leveraging genetic variants associated with JAK signaling as instrumental variables, MR enables the assessment of whether modulating JAK activity may confer benefits or potentially harmful effects in specific skin conditions. Comprehensive pharmacovigilance for drug safety indeed requires large-scale population studies and long-term data to capture rare or delayed adverse effects (Cohen and Reddy, 2023; Dagenais et al., 2022). However, Mendelian randomization analysis offers a valuable complementary perspective, enabling preliminary insights into potential adverse effects (Plenge et al., 2013; Ference et al., 2016). Importantly, genetically proxied inhibition is not equivalent to drug exposure: it reflects lifelong, modest on-target perturbation and does not capture dose-dependent, time-limited treatment effects, off-target toxicity, drug–drug interactions, or confounding by indication. Given key safety concerns reported for JAK-pathway inhibition—venous thromboembolism (including pulmonary embolism), serious infections (notably tuberculosis), and malignancies (e.g., lung and non-melanoma skin cancer)—we prespecified these outcomes as safety endpoints for MR/SMR and triangulated findings with trial evidence. By stratifying GWAS datasets into efficacy- and safety-related phenotypes, this study establishes a framework for precision evaluation of the therapeutic potential and adverse effects of JAK inhibitors, providing genetic insights to guide personalized treatment strategies (Figure 1).

## 2 Methods

### 2.1 Study design

Summary statistics from large-scale Genome-Wide Association Study (GWAS) and (expression quantitative trait locus) eQTL resources were integrated for Mendelian randomization analysis, using only publicly accessible datasets, as outlined in Table 1 and visualized in Figure 1. The GWAS datasets, which were classified into therapeutic diseases and potential adverse event-related outcomes, include data from the FinnGen study and the UK Biobank, ensuring no overlap of study populations. The UK Biobank study (discovery) 361,194 sample of British individuals and The FinnGen study (R11) (replication) 453,733 Finnish individuals. All contributing studies were approved by the respective institutional review boards, and informed consent was obtained from all participants. A systematic review was employed to contextualize and validate the Mendelian randomization findings by systematically assessing adverse events (AEs) highlighted as potentially significant by SMR analysis. An overview of the study design is presented in Figure 1.

**TABLE 1:** Data sources for immune-mediated skin diseases and adverse event-related outcomes in UKB and Finngen.

Study	Phenotype	Cases	Controls	GWASID
Skin Diseases				
Ukb (LeeLab)	Atopic dermatitis	2,110	404,817	ukb-saige-939
UK Biobank	Psoriasis	474	360,720	Ukb Round 2
UK Biobank	scleroderma	121	361,020	Ukb Round 2
UK Biobank	vitiligo	95	337,064	ukb-a-115
UK Biobank	Acute myocardial infarction	5,948	355,246	Ukb Round 2
UK Biobank	Urticaria	143	361,051	Ukb Round 2
UK Biobank	Systemic lupus erythematosus	415	360,726	Ukb Round 2
Finngen	Pemphigus	208	451,899	finngen_R11_L12_PEMPHIGUS
Finngen	Psoriasis	4,510	212,242	finn-b-L12_PSORIASIS
Finngen	scleroderma	450	423,041	finngen_R11_L12_LOCALSCLERODERMA
Finngen	Systemic lupus erythematosus	777	423,041	finngen_R11_L12_LUPUS
Finngen	Urticaria	12,576	438,050	finngen_R11_L12_URTICARIA
Finngen	vitiligo	131	207,482	finn-b-L12_VITILIGO
Adverse Event-Related Outcomes				
UK Biobank	Herpes zoster	161	361,033	Ukb Round 2
UK Biobank	COPD related to chronic infections	477	360,717	Ukb Round 2
UK Biobank	Liver & bile duct cancer	350	372,016	ieu-b-4915
UK Biobank	Liver cell carcinoma	168	372,016	ieu-b-4953
UK Biobank	Lung cancer	2,671	372,016	ieu-b-4954
UK Biobank	Lymphomas	1,752	359,442	Ukb Round 2
UK Biobank	Malignant neoplasm of sigmoid colon	1,241	461,769	ukb-b-15775
UK Biobank	Malignant non-melanoma skin cancer	23,694	372,016	ieu-b-4959
UK Biobank	Melanoma skin cancer	3,751	372,016	ieu-b-4969
UK Biobank	Pulmonary embolism	2,118	359,076	Ukb Round 2
UK Biobank	Tuberculosis	489	91,298	Ukb Round 2
Finngen	Malignant neoplasm of pancreas	1,992	345,118	finngen_R11_C3_PANCREAS_EXALLC
Finngen	DVT of lower extremities	7,216	392,860	finngen_R11_I9_PHLETHROMBDVTLOW
Finngen	Haemmorrhoids and perianal venous thrombosis	9,749	329,381	finngen_R10_K11_HAEMORR
Finngen	Herpes zoster	6,268	435,872	finngen_R11_AB1_ZOSTER
Finngen	COPD related to chronic infections	702	433,110	finngen_R11_COPD_OPPORTUNIST_INFECTIONS
Finngen	Myocardial infarction	28,546	378,019	finngen_R11_I9_MI_STRICT
Finngen	Pulmonary embolism	11,269	441,336	finngen_R11_I9_PULMEMB
Finngen	Respiratory tuberculosis	638	450,922	finngen_R11_AB1_RESP_TUBERCU_CONF

The UK Biobank study (discovery) 361,194 sample of British individuals.

The FinnGen study (R11) (replication) 453733 Finnish individuals.

### 2.2 Selection of JAK1, JAK2, JAK3, and TYK2 instrumental variables

As summarized in Table 1, we employed cis-eQTL as genetic instruments to proxy exposure to JAK inhibitor target genes, including JAK1, JAK2, JAK3, and TYK2. Publicly accessible eQTL data from the eQTLGen Consortium (https://www.eqtlgen.org/) were analyzed to identify blood-derived SNPs with minor allele frequency above 1% and genome-wide significance (p < 5 × 10^−8^) in their association with target gene expression. We focused on cis-eQTL instruments (i.e., variants located in the vicinity of the target gene) to better reflect on-target perturbation and reduce the risk of horizontal pleiotropy. These variants were identified from eQTL summary statistics and correspond to single nucleotide polymorphisms (SNPs) within a ±1,000 kb cis-window around each JAK gene that affect gene expression.

A total of 22 SNPs were selected for JAK1, 657 SNPs for JAK2, 1 SNPs for JAK3, and 542 SNPs for TYK2. These variants were used to simulate the pharmacological inhibition of each JAK protein in the analysis of skin diseases.

Instrument validation and colocalization. For SMR analyses, we applied the heterogeneity in dependent instruments (HEIDI) test to distinguish pleiotropy from linkage disequilibrium; signals failing HEIDI were not interpreted as supporting a shared causal variant and were deprioritized or discussed cautiously.

Because our analyses included both target-based SMR (primarily single cis-eQTL instruments) and conventional MR settings with multiple instruments, we used method-appropriate approaches to evaluate robustness and potential horizontal pleiotropy. For SMR, we restricted instruments to cis-eQTLs to favor on-target perturbation and used the HEIDI test (and Bayesian colocalization where available) to distinguish signals consistent with a shared causal variant from those potentially driven by LD linkage or allelic heterogeneity. For targets with only a single available instrument (e.g., JAK3), multi-instrument pleiotropy-robust methods were not applicable; therefore, these results were interpreted cautiously.

### 2.3 Source of outcomes

We collected GWAS data for nine IMIDs (atopic dermatitis, alopecia areata, psoriasis, vitiligo, dermatomyositis, urticaria, systemic lupus erythematosus, pemphigus, and scleroderma) from the UK biobank (361,194 sample of British individuals) and the FinnGen study (R11, 453,733 Finnish individuals). All outcome GWAS summary statistics were obtained from publicly available resources, and the association models had been adjusted for covariates as specified by the original GWAS/biobank protocols.

UK Biobank received ethical permits from the Northwest Multi-centre Research Ethics Committee, the National Information Governance Board for Health and Social Care in England and Wales, Immune-Mediated Skin Diseases were diagnosed by codes from the ICD-9 and ICD-10, and self-reported information. Details of the outcome data sources and definitions are provided in Table 1. Some of the GWAS datasets used in this MR analysis were obtained from the Lee Laboratory for Statistical Genetics and Data Science (Seoul National University, Seoul, Republic of Korea; https://www.leelabsg.org/resources), with adjustments made for sex, birth year, genotyping batch, and the first four principal components.

The FinnGen project integrates germline genotype data from Finnish biobanks with nationwide health registry data on clinically defined outcomes, encompassing up to 453,733 participants. In this study, we utilized data from the R11 release of FinnGen (Simpson et al., 2020) to perform MR analyses. Immune-mediated skin disease outcomes were identified based on ICD-8, ICD-9, and ICD-10 diagnostic codes. Detailed information on GWAS sources for skin disease outcomes is provided in Table 1.

### 2.4 Data analysis

#### 2.4.1 Assumptions

The MR analyses relied on three core instrumental variable assumptions (Benucci et al., 2023): Relevance—the selected cis-eQTLs are strongly associated with the expression of the target gene (Luo et al., 2021); Independence—the genetic variants are independent of potential confounders that could influence both gene expression and disease risk; and (Chapman et al., 2022a) Exclusion restriction—the instruments affect the outcome only through the exposure (gene expression) and not via alternative biological pathways. These assumptions were supported by the use of significant cis-eQTLs (P < 5 × 10^−8^) within ±100 kb of each gene and by implementing the HEIDI test to detect potential pleiotropy or linkage. For sensitivity analyses, heterogeneity and leave-one-out tests were performed to assess the robustness of causal estimates.

### 2.5 SMR analysis

Summary-data-based Mendelian Randomization (SMR) was used to integrate eQTL data with GWAS summary statistics, evaluating whether the genetically predicted expression levels of JAK1, JAK2, JAK3, and TYK2 were associated with the risk of the selected skin diseases. This approach enables the exploration of potential causal links between gene expression and disease susceptibility. We conducted SMR analysis using the SMR software (version 1.03; https://cnsgenomics.com/software/smr/#Overview) (Bieber et al., 2021), which integrates the Heterogeneity in Dependent Instruments (HEIDI) test to differentiate true pleiotropic effects from linkage. The HEIDI test was further employed to evaluate whether the detected associations were attributable to linkage disequilibrium (LD). A p-value < 0.01 was considered indicative of linkage, and those associations were excluded from further analysis. Missing or ambiguous SNPs were excluded at harmonization. Multiple testing was corrected by Bonferroni adjustment across all target–outcome pairs.

### 2.6 Tissue-specific SMR analyses using GTEx v8 (45 tissues)

#### 2.6.1 Tissue-specific SMR analyses (GTEx v8)

To evaluate tissue context and assess whether key target–outcome associations were consistent across relevant tissues, we performed tissue-specific SMR analyses using cis-eQTL resources from the Genotype-Tissue Expression (GTEx) project (v8). We used the GTEx v8 cis-eQTL summary data in BESD-lite (.lite) format across 45 tissues and tested genetically proxied expression of JAK1, JAK2, JAK3, and TYK2 against each study outcome. For each gene, we restricted instruments to cis-acting variants within ±1,000 kb of the gene region and applied the SMR framework using the top cis-eQTL per probe as the primary instrument. Where SMR associations were observed, we applied the HEIDI test (when available) as a heterogeneity check to distinguish signals consistent with a shared causal variant from those potentially driven by linkage disequilibrium. Tissue-specific results were used to support biological plausibility (e.g., skin-relevant tissues for dermatologic outcomes) and to triangulate findings obtained from whole-blood eQTL resource.

#### 2.6.2 Bayesian colocalization (COLOC)

For SMR-prioritized associations with available harmonized regional summary statistics, we performed Bayesian colocalization (COLOC) to assess whether eQTL and GWAS signals likely share a causal variant rather than reflect LD-driven linkage. We report posterior probabilities for PP.H0–PP.H4 and use PP.H4 as the primary indicator of colocalization support. Full COLOC results are provided in Supplementary Table S4.

### 2.7 Systematic review of safety data of clinical drug trials on JAK inhibitors

#### 2.7.1 Search strategy, eligibility, and outcomes

We searched PubMed from inception to 30 June 2024 (human studies only) using terms for immune-mediated dermatologic diseases and JAK inhibition (class term and individual agents). We additionally screened ClinicalTrials.gov and reference lists of eligible reports. The full search strategy is provided in the Supplementary Figure S1.

We included phase 3 randomized controlled trials of JAK inhibitors for dermatologic indications with regulatory approval or pending approval (US FDA/EU/Japan), comparing placebo or an active control. We excluded non-comparative studies, observational designs, case reports, and reviews.

Guided by a prespecified Population, Intervention, Comparison, and Outcome (PICO) framework, we extracted safety outcomes with emphasis on the four adverse events highlighted by our MR/SMR analyses: tuberculosis (TB), malignant non-melanoma skin cancer (NMSC), lung cancer (LC), and pulmonary embolism (PE). When trials reported broader composite endpoints (e.g., serious infections, malignancy categories, or VTE), we extracted the corresponding components where available.

#### 2.7.2 Data extraction and risk-of-bias assessment

Two reviewers (YPZ. And QDL) independently screened records, assessed full texts, and extracted data using a standardized form; discrepancies were resolved by consensus. Risk of bias was evaluated using the Cochrane Risk of Bias tool, with detailed judgments reported in Supplementary Table S6.

### 2.8 Statistical analysis

The validity of each SNP as an instrumental variable was assessed using the F-statistic, where values greater than 10 indicated sufficient instrument strength. To confirm the robustness of the findings, sensitivity analyses such as the leave-one-out approach were performed to check for potential influence from individual SNPs. All Mendelian randomization analyses were carried out in R (version 4.3.2) with the *TwoSampleMR* and *SMR* packages, while statistical analyses for the systematic review were completed using SPSS (version 28.0.1.1, IBM Corporation).

## 3 Results

### 3.1 SMR identified immune-mediated skin disease outcomes associated with JAK2 and TYK2 inhibition in UK biobank and FinnGen

#### 3.1.1 JAK1

**FIGURE 2:**
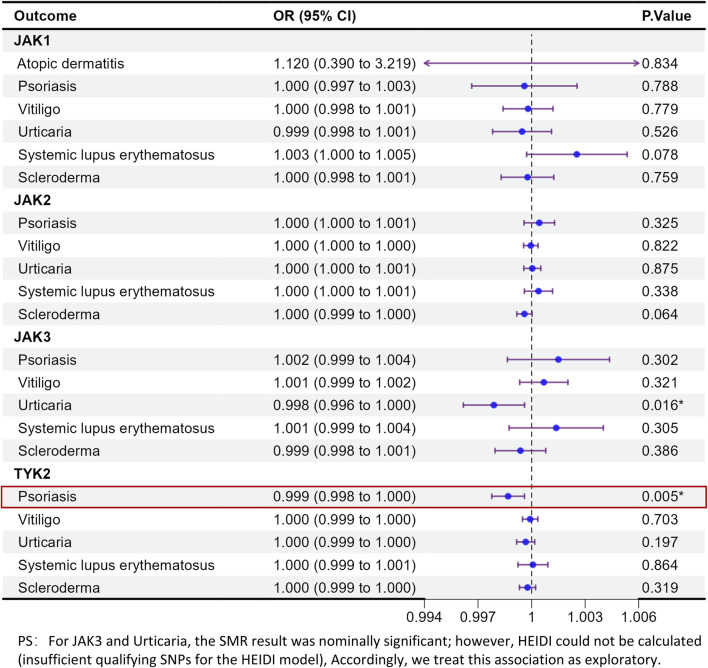
MR analysis of the health effects of JAK inhibition on Immune-Mediated Skin Diseases outcome in UK biobank.

**FIGURE 3:**
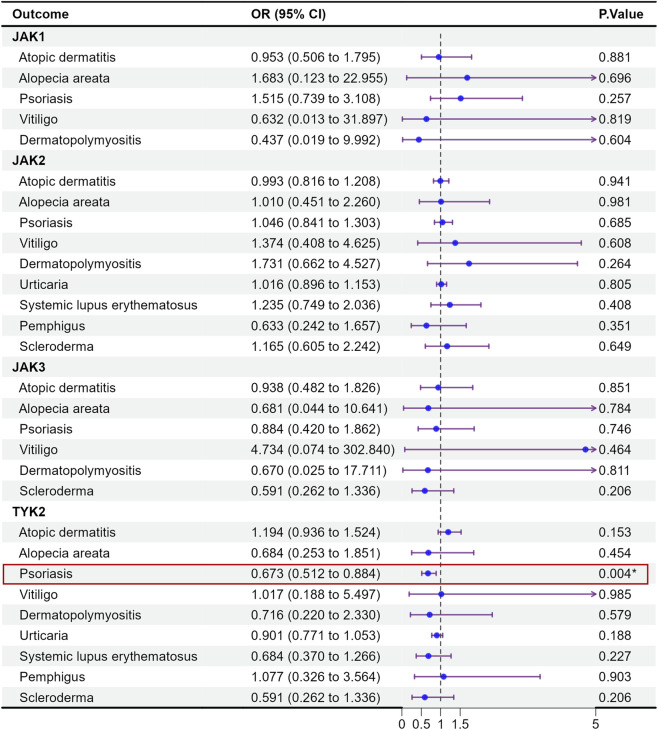
MR analysis of the health effects of JAK inhibition on Immune-Mediated Skin Diseases outcome in FinnGen (Replication).

A total of 22 eQTL SNPs were selected for JAK1. For some skin disease outcomes, there were insufficient SNPs for analysis. Among the six skin conditions that were analyzable—atopic dermatitis, psoriasis, vitiligo, urticaria, systemic lupus erythematosus, and scleroderma—no statistically significant genetic causal relationships with JAK1 were observed. The results from both the UK Biobank and Finnish datasets were consistent (Figures 2, 3).

#### 3.1.2 JAK2

No statistically significant genetic causal relationships with JAK2 and all the IMIDs outcomes were observed, including atopic dermatitis, alopecia areata, psoriasis, vitiligo, dermatomyositis, urticaria, systemic lupus erythematosus, pemphigus and scleroderma.

#### 3.1.3 JAK3

In the SMR analysis, expression of the JAK3 gene in blood significantly showed a geneticrelationship for urticaria (OR = 0.998, 95% CI = 0.996–1.000, p = 0.016). However, HEIDI could not be calculated (insufficient qualifying SNPs for the HEIDI model), Accordingly, we treat this association as exploratory. No significant associations were found for atopic dermatitis, alopecia areata, psoriasis, vitiligo, dermatomyositis, and scleroderma (p > 0.05 for all).

#### 3.1.4 TYK2

In the UK biobank dataset, expression of the TYK2 gene in blood was significantly associated with psoriasis (OR = 0.999, 95% CI = 0.998–1.000, p = 0.005). Furthermore, consistent directional associations were observed in outcome data from FinnGen studies (OR = 0.673, 95% CI = 0.512–0.884, p = 0.004). No significant associations were found for atopic dermatitis, alopecia areata, vitiligo, dermatomyositis, urticaria, systemic lupus erythematosus, pemphigus, and scleroderma (p > 0.05).

Using GTEx v8 tissue-specific cis-eQTL summary data from skin/lung-relevant tissues, we further evaluated whether TYK2 expression–outcome associations were supported in a tissue context. In these analyses, TYK2 expression showed a consistent inverse association with psoriasis, including the UK Biobank psoriasis dataset (OR = 0.998, 95% CI = 0.997–0.999, p = 0.003; HEIDI p = 0.465) and the FinnGen psoriasis dataset OR = 0.530, 95% CI = 0.375–0.751, p = 3.47 × 10^−4^) (Supplementary Table S3).

### 3.2 Adverse events (infectious diseases, cardiovascular and thromboembolic events, and malignancies) associated with genetically proxied JAK inhibition

**FIGURE 4:**
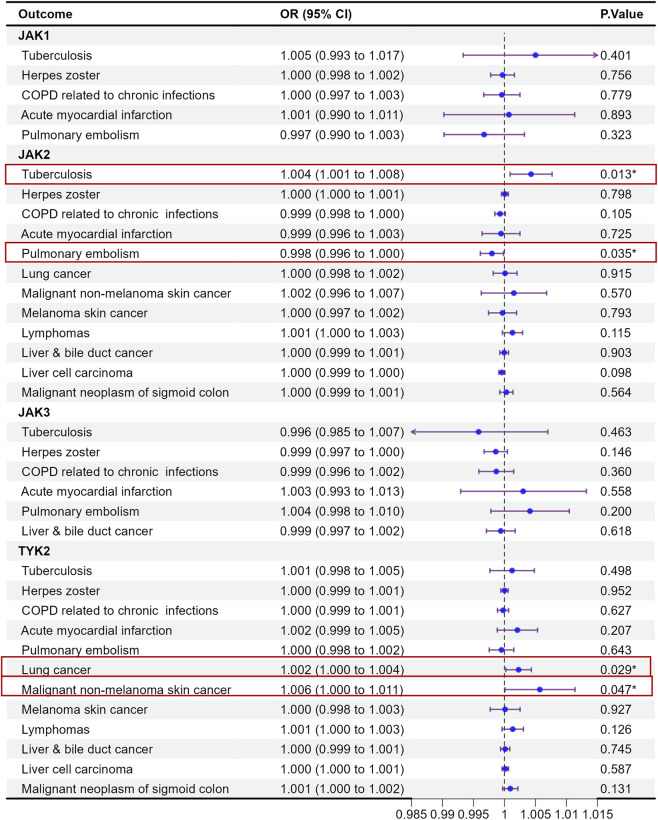
MR analysis of the health effects of JAK inhibition on adverse event-related outcomes in UK biobank.

**FIGURE 5:**
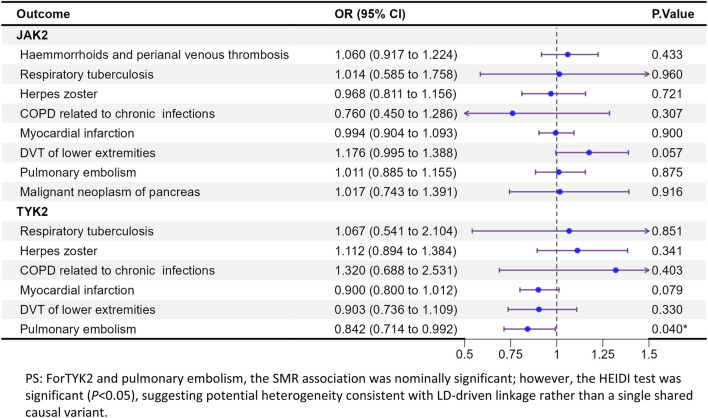
MR analysis of the health effects of JAK inhibition on adverse event-related outcomes in FinnGen (replication).

Based on adverse event (AE) records from previous clinical studies of JAK inhibitors, product labeling, and key safety warnings, we evaluated genetically proxied associations between JAK targets and several adverse outcome–related endpoints, including tuberculosis, herpes zoster, deep vein thrombosis of the lower extremities, acute myocardial infarction, pulmonary embolism, and opportunistic infections. These endpoints were selected *a priori* for clinical relevance and further prioritized because they overlapped with, or were supported by, SMR-identified signals observed in our analyses (Figures 4, 5).

Most results showed no statistically significant associations. Nevertheless, several signals merit attention, including JAK2–tuberculosis and pulmonary embolism (PE)–related associations observed in the SMR analyses. Specifically, in the UK Biobank, JAK2 showed a statistically supported association with tuberculosis (OR = 1.004, 95% CI = 1.001–1.008, p = 0.013) and with pulmonary embolism (OR = 0.998, 95% CI = 0.996–1.000, p = 0.035). In FinnGen, the TYK2 and PE association was also nominally significant; however, the HEIDI test was significant (P < 0.05), indicating heterogeneity that is more consistent with LD-driven linkage than a single shared causal variant. Overall, these findings highlight potential safety signals that warrant further investigation and triangulation with additional genetic and clinical evidence.

### 3.3 Review of RCTs on safety data of clinical drug trials on JAK inhibitors in immune-mediated skin diseases

A comprehensive review of randomized controlled trials (RCTs) on JAK inhibitors (JAK1, JAK2, JAK3, and TYK2) was conducted to evaluate their safety profiles, focusing on four key adverse events identified through Mendelian Randomization analysis: tuberculosis (TB), malignant non-melanoma skin cancer (NMSC), lung cancer, and pulmonary embolism (PE).

The initial search yielded 1,212 reports in Pubmed. After merging results and removing duplicates, and full-text screening, 35 RCTs (6 abrocitinib, 2 delgocitinib cream, 8 baricitinib, 7 upadacitinib, 4 ruxolitinib cream, 7 tofacitinib, and 1 ritlecitinib) met the inclusion criteria.

The main features of the included RCTs are outlined in Table 2. These studies mainly evaluated the therapeutic efficacy and safety profiles of JAK inhibitors in various immune-mediated skin disorders, including atopic dermatitis, psoriasis, alopecia areata, vitiligo, and psoriatic arthritis (Birney, 2022; Daghlas and Gill, 2023; Yarmolinsky et al., 2022; Yuan et al., 2023; Di Cesare et al., 2009; Ghoreschi et al., 2009; Catlett et al., 2022; Papp et al., 2018; Tehlirian et al., 2021; Tehlirian et al., 2022; Thaçi et al., 2022; Armstrong et al., 2021; Forman et al., 2020; Chen et al., 2022; K and aplan, 2020; Levy et al., 2015; Papp et al., 2021; Guttman-Yassky et al., 2021; Kennedy Crispin et al., 2016; Lee et al., 2018; Liu et al., 2018; Rosmarin et al., 2020; Mumford et al., 2020; Muddebihal et al., 2023; Alexander et al., 2021; Szekanecz et al., 2024; Khoo et al., 2020; Strober et al., 2023). In line with the genetically prioritized safety signals (SMR results), we prespecified four adverse events—pulmonary embolism, tuberculosis, lung cancer, and malignant non-melanoma skin cancer—for targeted extraction and synthesis. For each trial, adverse event data were extracted independently by two reviewers using a standardized form (event definition, number of events, denominator/exposure time when available, and comparator), and were cross-checked against the risk-of-bias assessment. Analysis of the four major adverse events was conducted, and the results are presented in Table 3.

**TABLE 2:** Baseline characteristics of included randomized clinical trials.

Source	Trial name	Disease condition	Intervention	JAKI No.	PBO/AC No.	Total	Follow-up, mo	Mean age, y	Male, %
King et al, 2022	BRAVE-AA1	Alopecia areata	Baricitinib	465	189	654	9	37	39.3
King et al, 2022	BRAVE-AA2	Alopecia areata	Baricitinib	390	156	546	9	37	39.3
King et al, 2023	ALLEGRO-2b/3	Alopecia areata	Ritlecitinib	587	131	718	12	33.8	37.9
Bieber et al, 2021	JADE Compare	Atopic dermatitis	Abrocitinib	464	374	838	4	37.9	50.4
Bieber et al, 2022	BREEZE-AD4	Atopic dermatitis	Baricitinib	370	93	463	4	38.4	62.5
Blauvelt et al, 2021	JADE REGIMEN	Atopic dermatitis	Abrocitinib	531	267	798	3	28.7	55
Blauvelt et al, 2021	Heads Up	Atopic dermatitis	Upadacitinib	348	344	692	4	36.7	54.5
Eichenfield et al, 2021	JADE Teen	Atopic dermatitis	Abrocitinib	96	189	285	12	15	50.9
Guttman-Yassky et al, 2021	Measure Up 1	Atopic dermatitis	Upadacitinib	566	281	847	4	34	54
Guttman-Yassky et al, 2021	Measure Up 2	Atopic dermatitis	Upadacitinib	558	278	836	4	33.6	56
Katoh et al, 2023	Rising Up	Atopic dermatitis	Upadacitinib	182	90	272	6	35.6	75.8
Nakagawa et al, 2020	Adults: QBA4-1	Atopic dermatitis	Delgocitinib cream	106	52	158	4	31.7	62
Nakagawa et al, 2021	Peds: Part 1	Atopic dermatitis	Delgocitinib cream	69	68	137	4	8.3	51.1
Papp et al, 2021	TRUE-AD1	Atopic dermatitis	Ruxolitinib cream	505	126	631	2	32	38
Papp et al, 2021	TRUE-AD2	Atopic dermatitis	Ruxolitinib cream	494	124	618	2	33	38.5
Reich et al, 2020	BREEZE-AD7	Atopic dermatitis	Baricitinib	220	108	328	4	33.8	66
Reich et al, 2021	AD Up	Atopic dermatitis	Upadacitinib	597	304	901	4	34	62
Riech et al, 2022	JADE DARE	Atopic dermatitis	Abrocitinib	365	362	727	6.5	36.6	53
Silverberg et al, 2020	JADE-MONO-2	Atopic dermatitis	Abrocitinib	313	78	391	3	35.1	58.6
Simpson et al, 2020	JADE MONO-1	Atopic dermatitis	Abrocitinib	310	77	387	3	54.8	32.5
Simpson et al, 2020	BREEZE-AD1	Atopic dermatitis	Baricitinib	375	249	624	4	35.8	62.7
Simpson et al, 2020	BREEZE-AD2	Atopic dermatitis	Baricitinib	370	244	614	4	34.5	62
Simpson et al, 2021	BREEZE AD5	Atopic dermatitis	Baricitinib	292	146	438	4	40	49
Torrelo et al, 2023	BREEZE-AD-PEDS	Atopic dermatitis	Baricitinib	361	122	483	4	12	50.7
Bachelez et al, 2015	NA	Psoriasis	Tofacitinib	659	442	1101	3	44.3	71.1
Papp et al, 2015	OPT Pivotal 1	Psoriasis	Tofacitinib	723	177	900	4	45.8	71.4
Papp et al, 2015	OPT Pivotal 2	Psoriasis	Tofacitinib	763	196	959	4	45.4	67.6
Zhang et al, 2017	NA	Psoriasis	Tofacitinib	178	88	266	12	41.1	72.9
Gladman et al, 2017	OPAL Beyond	Psoriatic arthritis	Tofacitinib	263	131	394	6	49.9	44.7
Leng et al, 2023	NA	Psoriatic arthritis	Tofacitinib	136	68	204	6	44.8	59.3
McInnes et al, 2021	SELECT-PSA 1	Psoriatic arthritis	Upadacitinib	852	852	1704	3	50.6	46.3
Mease et al, 2017	Opal Broaden	Psoriatic arthritis	Tofacitinib	211	211	422	12	48	44.6
Mease et al, 2021	SELECT-PSA 2	Psoriatic arthritis	Upadacitinib	429	212	641	6	53.4	54.3
Rosmarin et al, 2022	TRUE-V1	Vitiligo	Ruxolitinib cream	221	109	330	6	40.2	43.5
Rosmarin et al, 2022	TRUE-V2	Vitiligo	Ruxolitinib cream	228	115	344	6	38.9	49.9

**TABLE 3:** Summary results of the reported cases of the four AE-related outcome identified by SMR in clinical trial (N = number of patient reported).

Trail name	Source	JAKI				Placebo			
		TB	NMSC	LC	PE	TB	NMSC	LC	PE
AD Up	Reich et al, 24 2021	0	1	0	0	0	0	0	0
BREEZE-AD4	Bieber et al, 27 2022	0	4	0	0	0	0	0	0
Heads Up	Blauvelt et al, 22 2021	0	6	0	4	0	0	0	0
JADE REGIMEN	Blauvelt et al, 20 2021	0	1	0	0	0	0	0	0
JADE DARE	Riech et al, 19 2022	0	1	0	0	0	0	0	0
Measure Up 1	Guttman-Yassky et al, 23 2021	0	2	0	1	0	0	0	0
Measure Up 2	Guttman-Yassky et al, 23 2021	0	0	0	0	0	0	0	1
NA	Bachelez et al, 44 2015	0	4	0	0	0	0	0	0
Opal Broaden	Mease et al, 36 2017	0	1	0	0	0	0	0	0
OPT Pivotal 1	Papp et al, 32 2015	0	2	0	0	0	0	0	0
SELECT-PSA 1	McInnes et al, 34 2021	0	1	0	1	0	0	0	0
SELECT-PSA 2	Mease et al, 35 2021	0	2	0	0	0	0	0	0
NA	Leng et al, 38 2023	1	0	1	0	0	0	0	0
NA	Zhang et al, 33 2017	0	0	2	0	0	0	0	0
ALLEGRO-2b/3	King et al, 42 2023	0	0	0	1	0	0	0	0

TB, Tuberculosis; NMSC, Non- melanoma skin cancer; LC, Lung cancer; PE, Pulmonary embolism; N denotes the number of patients reported in the adverse-event (safety) dataset for each trial/arm.

#### 3.3.1 Tuberculosis (TB)

Only one RCTs reported lung neoplasm with latent TB as a potential adverse event of JAK inhibitor treatment, in a patient treated with Tofacitinib 5 m g BID.

#### 3.3.2 Malignant non-melanoma skin cancer (NMSC)

Among the RCTs included in the systematic review, 11 trials reported individual cases of NMSC without statistically significant risk-related outcomes. Notably, in the extended dataset (including long-term All-bari atopic dermatitis studies) (Simpson et al., 2020), a total of 6 NMSC cases were reported (incidence rate [IR] = 0.26/100 patient-years): these included 3 cases of basal cell carcinoma (all in the 2 mg dose group), 2 cases of Bowen’s disease (both in the 4 mg dose group), and 1 case of keratoacanthoma (in the 4 mg dose group)

#### 3.3.3 Lung cancer

Two randomized controlled trials identified lung cancer as an uncommon adverse event. In the study by Leng et al. involving Chinese patients with active psoriatic arthritis treated with tofacitinib (Khoo et al., 2020), a single case of malignant lung neoplasm was observed in a participant with a 40-year smoking history, chronic obstructive pulmonary disease, latent tuberculosis, and a pre-existing right lung nodule. Another randomized trial on plaque psoriasis treated with tofacitinib reported two lung cancer cases (Thaçi et al., 2022). One patient receiving tofacitinib 10 mg twice daily developed small-cell lung cancer, while another receiving 5 mg twice daily was diagnosed with metastatic lung adenocarcinoma. Both individuals had a prior history of smoking, suggesting a potential link between smoking and the occurrence of these malignancies.

#### 3.3.4 Pulmonary embolism (PE)

Four RCTs evaluated the risk of pulmonary embolism, including the ALLEGRO-2b/3, SELECT-PSA 1, Measure Up 2 and Heads Up trail. In the Heads-Up trail (Bieber et al., 2021), one venous thromboembolic event (PE) occurred in a 51-year-old female treated with 4 mg of baricitinib (IR = 0.38). The patient had risk factors including concomitant use of oral contraceptives and a history of smoking. Treatment was halted, leading to the patient’s recovery. In the extended analysis, another pulmonary embolism (PE) case was identified in the 4 mg group (IR = 0.40), occurring in a 61-year-old man with age as the only notable risk factor. Baricitinib therapy was subsequently stopped, and the patient showed signs of recovery. Across all studies (All-bari dataset), the incidence rate (IR) for PE was 0.09 per 100 patient-years, with two reported cases.

## 4 Discussion

This study systematically analyzes the genetic causal relationships between drug targets of currently available JAK inhibitors and IMIDs. It also explores the potential causal links between JAK targets and associated adverse effects, providing a comprehensive assessment of both therapeutic potential and safety concerns. Theoretically, some drug effects of JAK inhibitors are pathway-related, particularly those involving inflammatory factors and signaling networks. Others, however, are tied to the functional loss or specific genetic variations of the target itself. Results from Mendelian Randomization (MR) analysis primarily reflect the latter—effects due to inherent functional changes or genetic variations in the target (Birney, 2022). Consequently, findings from MR analyses warrant careful consideration, as they provide insights into potential drug impacts directly tied to genetic predispositions, rather than broader pathway effects (Daghlas and Gill, 2023). There have been numerous similar studies in the past that employed Mendelian Randomization (MR) to analyze drug targets in relation to both efficacy and adverse effects (Yarmolinsky et al., 2022; Yuan et al., 2023). A recent phenome-wide MR study supported TYK2 inhibition as a potential treatment for psoriasis, while highlighting the need for pharmacovigilance regarding possible adverse effects and cancer-related signals. (Yuan et al., 2023). The strengths of our study lie in its systematic focus on IMIDs and its exploration of potential indications for JAK inhibitors. Additionally, by leveraging high-quality datasets from the UK Biobank (UKB) and Finnish cohorts, the study provides valuable insights into adverse events (AEs) that may warrant further attention. Finally, the results of the Mendelian genetic analysis were validated by the findings from the systematic review.

### 4.1 Therapeutic efficacy of JAK inhibitors in IMIDs through the lens of mendelian randomization analysis

This study provides genetic evidence supporting the use of JAK3 inhibitors in the treatment of urticaria and TYK2 inhibitors in psoriasis. Psoriasis is a chronic inflammatory skin disorder characterized by the overactivation of the IL-23/Th17 axis, which leads to the proliferation of keratinocytes and inflammation (Di Cesare et al., 2009). TYK2, part of the JAK family, is essential for transmitting IL-23 and type I IFN signals, both of which are central to the development of psoriasis (Ghoreschi et al., 2009). Our genetic findings strongly support the use of TYK2 inhibitors in psoriasis, consistent with clinical evidence from trials of selective TYK2 inhibitors like deucravacitinib (Catlett et al., 2022). These inhibitors have demonstrated notable effectiveness in alleviating disease severity, reflected by improvements in Psoriasis Area and Severity Index (PASI) scores. (Catlett et al., 2022; Papp et al., 2018; Tehlirian et al., 2021; Tehlirian et al., 2022; Thaçi et al., 2022). Our findings reinforce the growing evidence that JAK inhibitors, particularly TYK2, hold therapeutic potential for specific IMIDs such as psoriasis (Papp et al., 2018; Armstrong et al., 2021; Forman et al., 2020).

However, the genetic causal association between JAK3 and urticaria requires further clinical validation to assess its therapeutic impact accurately. Currently, no relevant studies have been conducted. JAK3, on the other hand, plays a key role in lymphocyte development and function, as it is involved in signaling pathways mediated by the common gamma chain of cytokine receptors (e.g., IL-2, IL-7, IL-4, IL-9, IL-21and IL-15) (Chen et al., 2022). Currently, the treatment options for urticaria are limited (K and aplan, 2020). Medications such as cyclosporine and omalizumab can be used, but there is still a need to explore more effective drugs. JAK3 inhibitors are one potential option. Given JAK3’s role in immune signaling, specifically through cytokines involved in inflammation, JAK3 inhibition might offer a promising approach in managing urticaria, pending further clinical exploration. Currently, peficitinib and tofacitinib are the inhibitors available on the market that partially target JAK3. In the future, more specific JAK3 inhibitors may be developed.

Of course, the therapeutic efficacy of JAK inhibitors for skin diseases ultimately depends on clinical trial data. Numerous related studies are currently underway, focusing on JAK inhibitors’ effects on various skin conditions. Skin diseases that may show promising results include atopic dermatitis (Simpson et al., 2020; Levy et al., 2015; Papp et al., 2021; Guttman-Yassky et al., 2021), alopecia areata (Kennedy Crispin et al., 2016; Lee et al., 2018; Liu et al., 2018), and vitiligo (Rosmarin et al., 2020; Mumford et al., 2020). It is also essential to interpret negative findings with caution; a lack of statistically significant genetic associations does not imply that a JAK pathway inhibitor lacks therapeutic value for a particular condition. This is due to the broad and complex roles JAK pathways play across different immune functions (Muddebihal et al., 2023). Thus, while genetic evidence offers insights into likely therapeutic targets, the efficacy of JAK inhibitors across various conditions must still be verified through rigorous clinical studies. RCTs for these inhibitors have demonstrated efficacy in other diseases, such as rheumatoid arthritis, atopic dermatitis, and graft-versus-host disease (Alexander et al., 2021).

### 4.2 Adverse effects of JAK inhibitors through the lens of mendelian randomization analysis and systemic review

The side effects of a new drug may require a large and heterogeneous sample to be fully revealed. In such cases, Mendelian Randomization (MR) offers a valuable approach by providing genetic insights into potential adverse effects, allowing us to remain vigilant about possible safety concerns even before large-scale observational data are available. Importantly, MR/SMR identifies genetically supported associations consistent with target perturbation; however, these signals should not be interpreted as definitive proof of pharmacologic causation. This genetic analysis helps identify risks that might otherwise require extensive clinical data to uncover, supporting proactive safety assessments.

Currently, the safety data for JAK inhibitors primarily comes from studies on rheumatoid arthritis (RA), indicating that they are relatively safe. However, there are some important considerations to note, including not only the risk of major adverse cardiovascular events (MACEs) and cancers, but also venous thromboembolism and severe infections (Szekanecz et al., 2024). For patients with IMIDs, however, the potential adverse effects may differ due to variations in patient backgrounds. Factors such as the presence of comorbidities, prior treatments, and the disease’s immunological nature could influence the risk profile, necessitating careful monitoring and a personalized approach to safety assessments in this patient population.

In light of these situations, we conducted Mendelian randomization analysis on JAK targets and various potential adverse effect diseases in IMIDs patients. We observed statistically supported genetic associations (genetically proxied target perturbation signals) between both JAK2 and TYK2 and the risk of pulmonary embolism. For SMR-derived signals, we applied the HEIDI test as a linkage-versus-pleiotropy check; only signals with non-significant HEIDI (no evidence of heterogeneity) were prioritized for interpretation as being consistent with a shared causal variant, whereas signals failing HEIDI were deprioritized and discussed cautiously as potentially LD-driven.

Concordance and discordance with trial evidence. To make the comparison explicit, we triangulated the genetic signals with trial-reported adverse events in IMID RCTs and external clinical/regulatory evidence, and we observed both concordant and discordant patterns. Similarly, recent clinical research and regulatory assessments have indicated an elevated risk of venous thromboembolism (VTE), including pulmonary embolism (PE), associated with the use of JAK inhibitors, particularly tofacitinib and baricitinib (Khoo et al., 2020). Thus, the PE signal for JAK2 (and to a lesser extent TYK2) is directionally concordant with broader post-marketing/RA safety concerns regarding VTE/PE. And in our systemic review in IMDs, across all studies (All-bari dataset), the incidence rate (IR) for PE was 0.09 per 100 patient-years, with two reported cases. It appears to be lower than in clinical trials for other indication. In contrast, TYK2 inhibitors have fewer reported cases of pulmonary embolism (PE) (Strober et al., 2023; Mease et al., 2022; Morand et al., 2023); However, the relatively short duration of available studies necessitates caution. Given the rarity of PE events and limited follow-up in RCTs, absence of a strong trial signal does not exclude risk; rather, our genetic findings should be viewed as complementary safety signals warranting continued surveillance and longer-term studies. Biologic plausibility and alternative explanations. The PE/VTE signal is biologically plausible because JAK2 is centrally involved in hematopoiesis and cytokine signaling that can influence inflammatory-thrombotic pathways; nevertheless, alternative explanations include LD-driven associations (for SMR signals failing HEIDI), outcome misclassification across datasets, and differences in baseline thrombotic risk between RA and dermatologic IMID populations. Continued monitoring and longer-term research are essential to fully understand the thromboembolic risks associated with TYK2 inhibition.

Based on our findings, it appears that JAK2 and tuberculosis also warrants attention. Our results suggest a genetically supported association between JAK2 and TB. These findings highlight the need for vigilant TB screening and prophylaxis in patients receiving JAK2 inhibitors, particularly in regions with a high prevalence of tuberculosis. Clinical studies on JAK2 inhibitors for rheumatoid arthritis have also reported an elevated incidence of tuberculosis (Winthrop et al., 2016). This suggests that the need for careful screening and monitoring of patients at risk when using JAK2 inhibitors in clinical settings (Winthrop et al., 2020), However, in our systemic review in IMDs, no active tuberculosis was reported. This is a discordant pattern between genetic evidence and dermatology RCT reporting, which may be explained by trial exclusion of high-risk individuals, routine TB screening/prophylaxis protocols, short exposure windows, and limited statistical power to detect rare infections in IMID RCTs. Therefore, the absence of TB cases in the included RCTs should be interpreted as insufficient evidence rather than evidence of no risk.

In analyses of JAK targets and cancer associations, TYK2 showed a statistically supported genetic association with an increased risk of both malignant non-melanoma skin cancer and lung cancer. These findings should be interpreted cautiously as potential on-target safety signals rather than proof that TYK2 inhibitor treatment causes cancer, because genetic proxies reflect lifelong modest perturbation and do not capture dose-dependent, time-limited pharmacologic inhibition or off-target effects. This suggests TYK2 inhibition may influence pathways related to tumor development, underscoring the need for further research and vigilance regarding cancer risk when considering TYK2 inhibitors in clinical applications. Actually, JAK inhibitors, used primarily for chronic inflammatory conditions, have been linked to an elevated risk of certain cancers in recent studies. These include lung cancer and lymphoma, particularly in patients with existing risk factors like a history of smoking. This association was highlighted in a safety study for tofacitinib (Ytterberg et al., 2022), which demonstrated a higher cancer incidence compared to TNF inhibitors (e.g., etanercept or adalimumab) among patients with rheumatoid arthritis. Similar concerns have been extended to other JAK inhibitors, including baricitinib and upadacitinib. In our systemic review in IMDs, 11 trials reported individual cases of NMSC without statistically significant risk-related outcomes, and Two RCTs reported lung cancer as a rare adverse event. Given the low event counts, short follow-up, and cancer latency, RCT estimates for malignancy (and site-specific cancers) are often imprecise and should be interpreted with wide uncertainty.

Concordance/discordance interpretation for malignancy. Overall, the malignancy findings show partial concordance: malignancy is a recognized safety concern for JAK-pathway inhibition (supported by non-dermatology clinical/regulatory evidence), while dermatology IMID RCTs generally provide limited and imprecise estimates due to rare events and inadequate latency. Alternative explanations for discordant or null RCT signals include short trial duration relative to cancer latency, under-ascertainment, and heterogeneity across cancer subtypes (e.g., NMSC vs. internal malignancies), which may dilute site-specific risks. Given the background of skin diseases, the risk of non-melanoma skin cancer (NMSC) with the use of JAK inhibitors should be given greater attention. A thorough cancer risk assessment should be conducted prior to initiating treatment.

Summary of triangulation. Taken together, our MR/SMR results and systematic review suggest that PE/VTE, TB, and malignancy outcomes (NMSC and LC) merit prioritized monitoring during JAK/TYK2 inhibition. Where RCT evidence appears discordant or neutral, this is most plausibly attributable to limited event counts and follow-up rather than true absence of risk, underscoring the need for longer-term surveillance and adequately powered post-marketing studies.

### 4.3 Study limitations

Our study has several limitations. The genetic data used were predominantly from European populations, which may limit the generalizability of the findings to other ancestries. Although MR reduces confounding and reverse causation, it relies on key assumptions (instrument relevance, independence from confounders, and exclusion restriction). Residual horizontal pleiotropy or LD with nearby functional variants may still bias estimates, even though we prioritized cis instruments, applied LD control, and used SMR validation (e.g., HEIDI/colocalization where available).

Genetically proxied target perturbation does not equal pharmacologic inhibition. Genetic instruments typically reflect lifelong, modest changes in gene expression/activity, whereas drug effects are dose-dependent, time-limited, and may include off-target/class effects. Therefore, MR/SMR signals should be interpreted as on-target genetic evidence and potential safety signals rather than direct treatment-effect estimates. For JAK3, only a single independent cis-eQTL instrument was available under our selection criteria, which limits pleiotropy-robust and heterogeneity-based sensitivity analyses and therefore the JAK3-related estimates should be interpreted cautiously.

Finally, biases in GWAS resources may affect inference, including residual population stratification, selection/phenotype heterogeneity across biobanks, and differences between the source populations (UKB/FinnGen) and treated IMID patients. Our systematic review helps contextualize these signals, but rare outcomes (e.g., PE and site-specific malignancies) and cancer latency require larger samples and longer follow-up than most dermatology RCTs provide.

## 5 Conclusion

This study provides robust genetic and multi-omics evidence supporting the use of JAK3 inhibitors in urticaria and TYK2 inhibitors in psoriasis. These findings are consistent with existing clinical research, reinforcing the therapeutic potential of these inhibitors as targeted treatments for autoimmune skin diseases.

By integrating Mendelian randomization and a systematic synthesis of clinical data, our study bridges large-scale population genomics with translational evidence, thereby illuminating both the efficacy and safety spectra of JAK inhibition. Notably, the results reveal potential safety signals, including the genetic causal relationships between JAK2 inhibition and pulmonary embolism or tuberculosis, and between TYK2 inhibition and pulmonary embolism, malignant non-melanoma skin cancer, and lung cancer.

Collectively, this integrative multi-omics framework provides a comprehensive evaluation of JAK inhibitors from both genetic and clinical perspectives. It highlights how multi-layered genomic information can inform risk stratification and guide precision therapeutic strategies in autoimmune dermatology.

## Data Availability

The datasets presented in this study can be found in online repositories. The names of the repository/repositories and accession number(s) can be found in the article/Supplementary Material.
