# Supplementary figures and images for: Genetic pharmacoepidemiology of JAK inhibitors in chronic immune-mediated skin diseases: implications for precision therapy and medication safety
Source: Front Pharmacol. 2026 Mar 19;17:1738089. doi: 10.3389/fphar.2026.1738089 (PMC13044065; doi:10.3389/fphar.2026.1738089)

Supplementary Figure 1. PRISMA flow

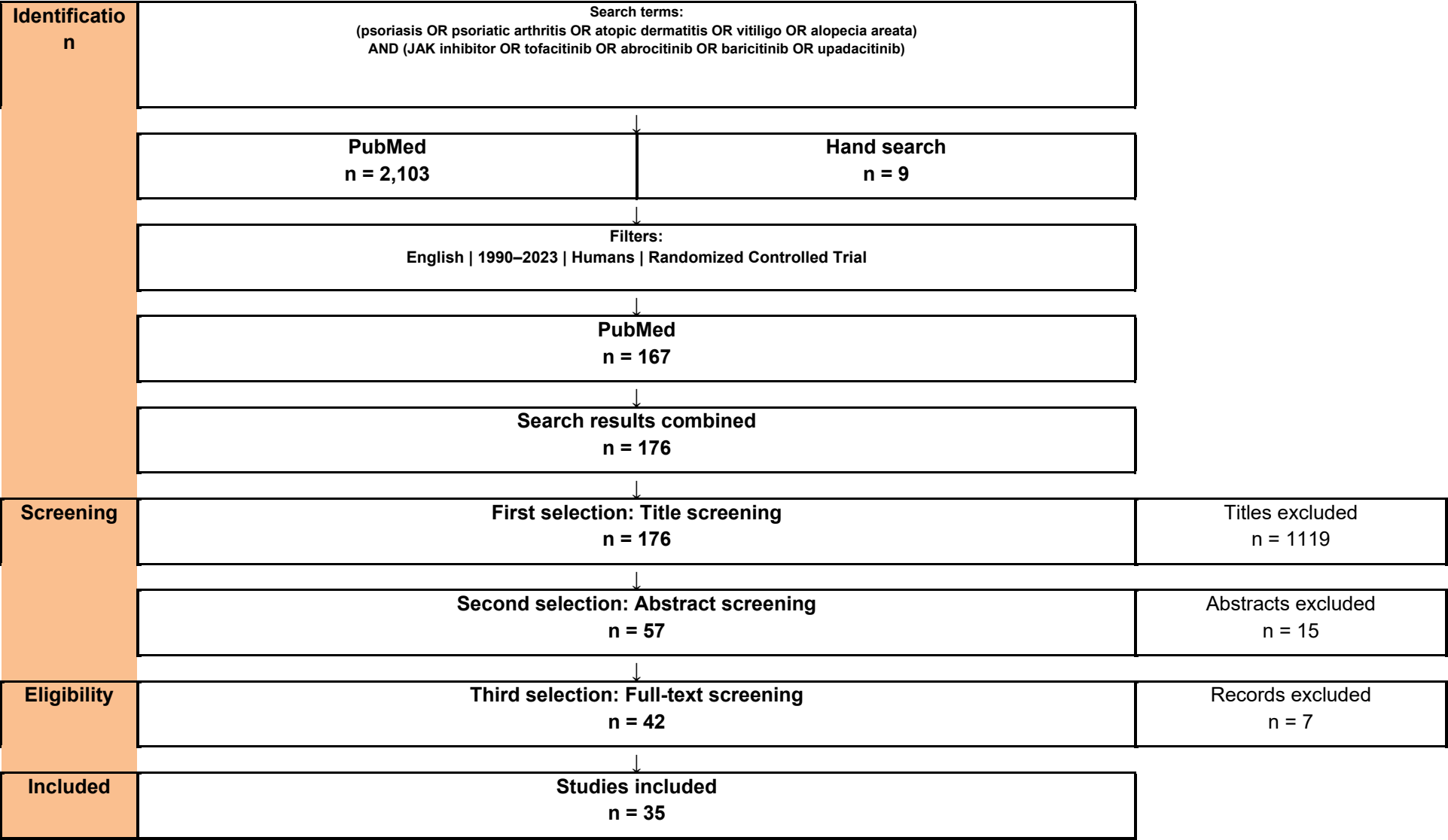

Supplement: Supplementary file 2 [file Image1.pdf]
